# Macrophage Function in the Pathogenesis of Non-alcoholic Fatty Liver Disease: The Mac Attack

**DOI:** 10.3389/fimmu.2019.02893

**Published:** 2019-12-12

**Authors:** Jarren R. Oates, Melanie C. McKell, Maria E. Moreno-Fernandez, Michelle S. M. A. Damen, George S. Deepe, Joseph E. Qualls, Senad Divanovic

**Affiliations:** ^1^Department of Pediatrics, University of Cincinnati College of Medicine, Cincinnati, OH, United States; ^2^Division of Immunobiology, Cincinnati Children's Hospital Medical Center and the University of Cincinnati College of Medicine, Cincinnati, OH, United States; ^3^Immunology Graduate Program, Cincinnati Children's Hospital Medical Center and the University of Cincinnati College of Medicine, Cincinnati, OH, United States; ^4^Division of Infectious Diseases, Cincinnati Children's Hospital Medical Center and the University of Cincinnati College of Medicine, Cincinnati, OH, United States; ^5^Department of Medicine, University of Cincinnati College of Medicine, Cincinnati, OH, United States; ^6^Center for Inflammation and Tolerance, Cincinnati Children's Hospital Medical Center, Cincinnati, OH, United States

**Keywords:** macrophage, metabolism, NAFLD, inflammation, cytokines

## Abstract

Obesity is a prevalent predisposing factor to non-alcoholic fatty liver disease (NAFLD), the most common chronic liver disease in the developed world. NAFLD spectrum of disease involves progression from steatosis (NAFL), to steatohepatitis (NASH), cirrhosis and hepatocellular carcinoma (HCC). Despite clinical and public health significance, current FDA approved therapies for NAFLD are lacking in part due to insufficient understanding of pathogenic mechanisms driving disease progression. The etiology of NAFLD is multifactorial. The induction of both systemic and tissue inflammation consequential of skewed immune cell metabolic state, polarization, tissue recruitment, and activation are central to NAFLD progression. Here, we review the current understanding of the above stated cellular and molecular processes that govern macrophage contribution to NAFLD pathogenesis and how adipose tissue and liver crosstalk modulates macrophage function. Notably, the manipulation of such events may lead to the development of new therapies for NAFLD.

## Introduction

The unabated obesity pandemic is directly linked with the incidence of non-alcoholic fatty liver disease (NAFLD). NAFLD afflicts ~35% of obese individuals worldwide (1, 2). Current epidemiological estimates suggest that NAFLD will soon surpass chronic hepatitis C infection as the leading cause of liver transplantation. Given the lack of effective therapies for NAFLD, costs of care and management of associated symptoms come with a considerable economic burden (3).

NAFLD spectrum of disease progresses from non-alcoholic fatty liver (NAFL) or hepatic steatosis, to non-alcoholic steatohepatitis (NASH), to cirrhosis and hepatocellular carcinoma (HCC). Steatosis is characterized by increased macrovesicular and microvesicular lipid droplet accumulation that occurs in more than 5% of hepatocytes (1, 2, 4). Approximately 25% of individuals afflicted with NAFL progress to NASH (1). NASH is characterized by hepatocellular ballooning, in part due to increased immune cell infiltration, activation, and proinflammatory cytokine production (4–6). These mechanisms coupled with others such as adipokine production and activation of endoplasmic reticulum stress and reactive oxygen species (ROS) promote hepatic fibrosis and progression to cirrhosis and HCC (7, 8).

The contribution of various immune cells in hepatic inflammation and the mechanisms that govern their migration to the liver, polarization, and inflammatory capabilities, in NAFLD progression represent an intense area of investigation. Here, we specifically focus on the contribution of macrophages to NAFLD pathogenesis. We review the landscape of underlying mechanisms that regulate macrophage effector functions and macrophage interplay with other immune cells/tissues/organs which collectively contribute to NAFLD progression.

## Current knowledge and Discussion

### Immune Responses in NAFLD

Dysregulated immune responsiveness is central to the development and progression of NAFLD (9, 10). In obesity, both liver resident (e.g., Kupffer cells, [KC], hepatic stellate cells, [HSC], hepatocytes) and infiltrating immune cells (e.g., neutrophils, dendritic cells [DC], natural killer [NK] cells, NKT cells, blood monocytes, T cells, B cells, and macrophages) contribute to NAFLD development and progression via systemic and tissue inflammatory mediator production (e.g., interleukin [IL]-17A, IL-6, tumor necrosis factor [TNF], IL-1β) (5, 11). Obesity-associated intestinal permeability and augmented circulating levels of inflammatory ligands (e.g., lipopolysaccharide [LPS]) (12) via activation of pattern recognition receptors (PRRs) on hematopoietic and non-hematopoietic cells activate multiple proinflammatory cascades that in unison promote liver injury (13). The contribution of PRRs to NAFLD progression has been reviewed in detail elsewhere (14, 15). PRR signaling in macrophages, also contributes to activation of adaptive immune responses, with macrophage-T cell interplay having a particularly important role in NAFLD progression (16). In this setting, activated, liver infiltrating T cells produce proinflammatory cytokines and amplify macrophage polarization and activation to in turn propagate overall hepatic inflammation, hepatocellular damage and hepatocyte release of damage associated molecular patterns (DAMPs). Cumulatively these processes fuel and support a chronic inflammatory state in the liver that is a hallmark of NAFLD progression. Due to the extent of various immune processes in NAFLD, here we selectively focus on the role of macrophage-mediated inflammation and their contribution to NAFLD pathology. The contribution of other immune cells (e.g., T cells, neutrophils, DC, NK cells, and NKT cells) and cytokines in NAFLD has been discussed elsewhere (14, 17, 18).

### Macrophage Recruitment to the Liver

Liver infiltration by inflammatory monocytes/macrophages is associated with NAFLD progression (19). Increased release of free fatty acid (FFA) by white adipose tissue (WAT) augments triglyceride synthesis and storage in hepatocytes and induces hepatocyte release of inflammatory mediators including proinflammatory cytokines and macrophage recruiting chemokines (e.g., CCL2, CXCL10) (20, 21). In addition to hepatocytes, HSCs, myofibroblast and macrophages themselves can also produce various chemokines (e.g., CCL2, CCL3, CCL4, CCL5, CCL8, and CXCL10) to fuel increased macrophage recruitment (22).

The contribution of hepatic macrophage recruitment to NAFLD pathogenesis is supported in part by increased systemic and hepatic chemokine levels in NAFLD progression in humans (23). One of the most widely explored pathways of recruiting inflammatory and fibrogenic monocytes to the injured liver is the CCR2/CCL2 axis (24). Pharmacological inhibition of CCR2 and genetic deletion of CCL2 reduced liver steatosis in obese mice (25–27). Additionally, use of CCR1-, CCR2-, CCR5-, and CCR8-deficient mice or pharmacological inhibition of these axes reduced hepatic macrophage infiltration, hepatic fibrosis, and hepatocellular damage in experimental models of chronic liver injury (28–32). Recent evidence also suggests that CXCR3-deficient mice are protected from macrophage infiltration and hepatocellular damage in obesity (33, 34). Further, CXCL10-deficient mice exhibit reduced NAFL (34). Despite its promising effects in animal models, targeting of the CCR2 axis in human NALFD using Cenicriviroc (CVC) did not impact hepatic lobular inflammation and only mildly improved fibrosis and decreased circulating levels of sCD14 (a marker of monocyte activation) (35). Characterization of the intrahepatic immune cells will be required to elucidate the effects of CVC on immune cells, monocyte and macrophage recruitment and fibrogenesis. The results of such could give insight to observed difference of effects of CVC on NAFLD progression between murine models and humans.

Like chemokines, inflammatory cytokines can also alter macrophage activation and tissue recruitment in NAFLD. For example, the IL-17A axis impacts macrophage recruitment in the liver (36), while IL-17RA depletion on macrophages ameliorates NAFLD severity (37). However, as IL-17A can also activate liver parenchymal cells to modulate macrophage recruitment. Thus, detailed studies focused on the role of IL-17 family members, their cognate receptors and cell specific expression in NAFLD pathogenesis are needed. The overall role of the IL-17 axis in NAFLD pathogenesis has been reviewed in detail elsewhere (18).

### Macrophage Subsets

KCs reside in the anatomical areas that receive venous blood from the gut including hepatic sinusoids, hepatic lymph nodes and portal tract (38). Approximately 80% of the liver blood supply comes from the gut via the portal vein (39). As such, KCs act as key sentinels of the gut-liver interface. Under homeostatic conditions, PRR signaling instructs KCs to govern liver immunity by maintaining tolerance to harmless immunogens and cellular debris while in parallel enabling them to mount a response against pathogenic invaders via phagocytosis, cytokine production and antigen presentation. Collectively, the adaptation to latter events is central to preventing dissemination of microbes into peripheral circulation (5, 39, 40). In obesity, increased intestinal permeability, trafficking of bacteria into the gut lumen and LPS sensing (2, 40, 41) fuel KC activation and alter their function (42). In this context, activated KCs favor inflammatory responses that contribute to NAFLD pathogenesis (13). *In vitro* treatment of KCs with FFAs (e.g., palmitate) promotes activation and secretion of inflammatory cytokines (e.g., IL-6, TNF, IL-1β) (43) while KC depletion *in vivo* protects from obesity-driven hepatic steatosis (13).

**Figure 1:**
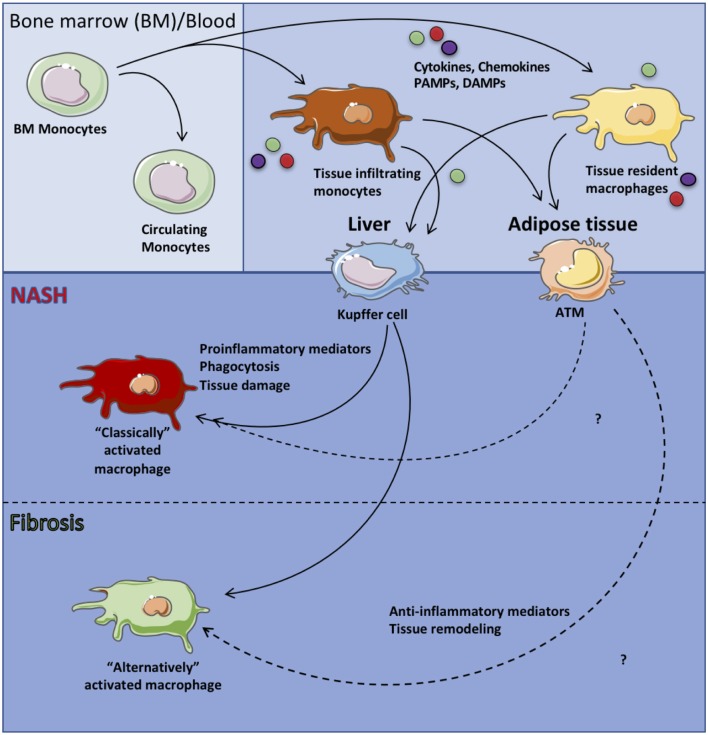
Macrophage subsets in health and disease. Circulating monocytes originating from the bone marrow are recruited to specific tissues and differentiate into tissue resident macrophages. In the context of systemic inflammation, circulating monocytes as well as tissue resident macrophages are activated by sensing of proinflammatory mediators (i.e., IL-6, TNF, IL-1β), chemokines and ROS or anti-inflammatory mediators (i.e., IL-10) leading to “classically” or “alternatively” activated tissue macrophages, respectively which then contribute to tissue pathology.

Common bone marrow myeloid progenitors give rise to granulocyte-macrophage progenitors (GMP) from which monocytes are derived. Upon egression from the bone marrow, and following hepatic inflammatory insult, circulating monocytes traffic to the liver. Once in the liver, in response to cytokines and various pathogen associated molecular patterns (PAMPs)/DAMPs, monocytes activate unique transcriptional profiles and differentiate into macrophages. In response to IFNγ or PRR signaling, recruited monocytes differentiate into “classically” activated macrophages that produce proinflammatory cytokines (e.g., IL-6, TNF, IL-1β, IL-12), drive liver recruitment of various immune cells and enhance the overall hepatic inflammation (5, 13, 44). Conversely, in response to either IL-4 or IL-13, tissue recruited monocytes differentiate into “alternatively” activated macrophages that produce anti-inflammatory and wound healing mediators (e.g., IL-8, MCP-1, IL-10) (38, 45, 46). The overall balance of “classically” and “alternatively” activated macrophages in the liver regulates hepatic inflammation, liver scarring and fibrosis. Targeting of inflammatory signaling pathways in macrophages via deletion of JNK, IKKβ, or Toll-like receptor (TLR) 4 is sufficient to reduce hepatic steatosis and inflammation (47–49). A brief summary of the above discussed processes is depicted in Figure 1.

### Macrophages and Proinflammatory Cytokine Production

Macrophage produced cytokines (e.g., IL-6, TNF, IL-1β) can directly target hepatocytes and promote steatosis, inflammation and hepatocellular damage (5). Systemic increase of these proinflammatory cytokines positively correlates with hepatocellular damage in humans and is recapitulated in NAFLD experimental mouse models (50, 51).

IL-6 is a multifunctional cytokine that regulates immune responses, acute phase reactions, hematopoiesis, and plays key roles in inflammation, host defense and tissue injury (52, 53). IL-6 stimulates hepatic lipogenesis (54), and is associated with obesity (55), impaired insulin signaling (56, 57), and altered insulin sensitivity by activating key steps in the insulin signaling pathway (58). IL-6 is also a biomarker of insulin resistance and cardiovascular diseases risk (50, 59, 60). In humans with NASH, there is a positive correlation between IL-6 expression in hepatocytes and the severity of NAFLD (61). IL-6-deficient mice display a milder NAFLD severity and antibody mediated IL-6 receptor (IL-6R) neutralization improved liver damage in mice fed methionine choline deficient (MCD) diet, despite enhanced steatosis (51, 62).

TNF stimulates hepatic fatty acid synthesis (FAS), increases serum triglyceride (TG) levels (63), stimulates very low density lipoprotein (VLDL) production from liver and contributes to impaired insulin signaling (64, 65). TNF also activates harmful proatherogenic pathways via the reduction of high-density lipoprotein (HDL)-cholesterol, elevated expression of cholesterogenic genes, accompanied by an increase in potentially harmful precholesterol metabolites, and suppression of cholesterol elimination (66). Thus, it is not surprising that TNF sensing by hepatocytes promotes hepatocyte cell death and hepatocyte proliferation (67), and as such directly contributes to NAFLD pathogenesis (68). Further, deletion of TNF in experimental mouse models of NAFLD correlates with decreased steatosis, fibrosis and improved glucose tolerance (69).

IL-1β promotes liver steatosis, inflammation and fibrosis via activation of the IL-1 receptor (IL-1R) signaling (70). IL-1β stimulates TG and cholesterol accumulation in hepatocytes and as such contributes to the development of hepatic steatosis (71). Mechanistically, IL-1β also promotes liver inflammation by inducing IL-6 production, upregulating ICAM-1 and neutrophil infiltration and accrual in the liver (72). IL-1R-deficient mice are protected from liver fibrosis (73). Hepatocyte-specific deletion of IL-1R attenuates liver injury in a model of acute liver disease (74). Whether similar effects are observed in animal models of NAFLD have not been examined. Further, IL-1R activates Myd88 signaling similar to TLRs (75). Thus, the role of IL-1R signaling in NAFLD warrants further investigation. In addition, blockade of IL-1 signaling with anakinra improved glycemic control in patients with T2D (76), suggesting the importance of inflammatory mediators in liver disease pathogenesis.

### Macrophages and ROS Production

Reactive oxygen species (ROS) production, a central antimicrobial effector function of macrophages, can be induced in part via macrophage sensing of proinflammatory cytokines. Macrophages generate ROS via numerous mechanisms including ER stress, mitochondrial damage and activation of nicotinamide adenine dinucleotide phosphate (NADPH) oxidases (NOXs). NOX2, also known as the phagocytic nicotinamide adenine dinucleotide phosphate NADPH oxidase, is constitutively associated with p22phox at the plasma membrane. PRR signaling in KCs and infiltrating macrophages causes complexing of NOX2 with other proteins (p67, P40, Rac GTPases) to generate superoxide, drive proinflammatory cytokine production (e.g., IL-6, TNF, IL-1β, transforming growth factor-β [TGFβ]) and promote hepatic steatosis, hepatocellular damage and fibrosis (43, 77, 78). NOX2-deficient mice display reduced macrophage-associated proinflammatory cytokine production, hepatic steatosis and fibrosis, and overall NAFLD severity in obesity (43, 79, 80). However, the underlying mechanisms regulating macrophage ROS production in NAFLD are not fully understood. Thus, in depth interrogation of the interplay between inflammatory cytokines and NADPH components on macrophage ROS production in NAFLD progression is needed.

### Macrophage Cellular Metabolism

**Figure 2:**
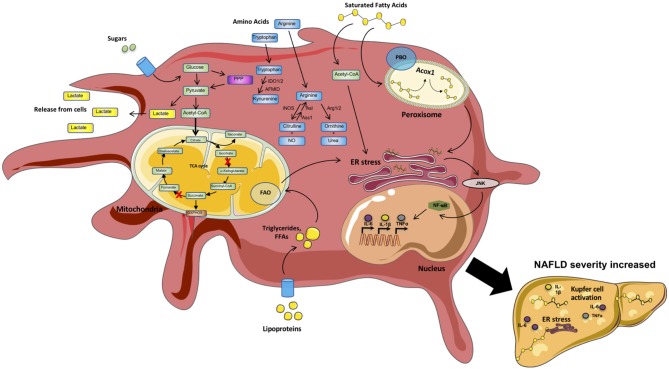
Metabolic processes within inflammatory macrophages. Macrophages are highly metabolically active cells. Their metabolic identity is impacted by inflammatory mediators. In contrast, specific metabolic pathways (Fatty acid synthesis [FAS], Glycolysis, Amino acid [AA] metabolism) regulate the type of mediators produced by these cells. During a proinflammatory state, inflammatory mediators (e.g., sugars, lipoproteins, saturated fatty acids [SFAs], cytokines [IL6, TNF]) trigger “classical” activation of circulating and tissue resident macrophages (e.g., Kupfer cells and adipose tissue macrophages [ATMs]). Circulating sugars are taken up and processed via glycolysis/TCA cycle. In addition, several intermediate metabolites, as well as amino acids L-arginine, L-tryptophan and glutamine, can impact macrophage effector functions. Sensing/uptake of excessive lipoproteins and SFAs activates the mitochondrial fatty acid oxidation (FAO) and peroxisomal fatty acid beta-oxidation (PBO) pathways to breakdown long chain and very long chain fatty acids, respectively. Excessive activation of these pathways triggers ER stress and signaling via JNK and NF-kB, resulting in amplified production of proinflammatory mediators. “Classical” macrophage activation shifts the cells toward preferential utilization of glycolytic pathways with altered enzyme activity within the tricarboxylic acid cycle (TCA) cycle generating more lactate and fast energy production in the form of ATP to generate inflammatory mediators (e.g., IL-6, TNF, IL1). Metabolism of tryptophan (L-TRP) and arginine (L-ARG) by macrophages regulates key immunologic processes. Cumulatively, these inflammatory mechanisms fuel the overall systemic and tissue inflammation, hepatocyte death, and fibrosis in turn amplifying NAFLD pathogenesis.

Metabolic pathways regulate immune cell function and inflammation (81, 82). Obesity alters cellular metabolism (83). In fact, both obesity-associated and inflammation-driven derangements in cellular metabolism are implicated in NAFLD progression (84–86). The full discovery of specific metabolic pathways and genes detrimental to NAFLD pathogenesis however remains an intense area of investigation. Macrophage-driven inflammation and resolution of inflammation are intricately linked to various metabolic pathways and several clinical phenotypes (i.e., insulin resistance, hyperlipidemia, etc.) (86, 87). Here we review metabolic pathways that contribute to macrophage-intrinsic inflammation and resolution in NAFLD. A brief summary of these processes is depicted in Figure 2 and Table 1.

**Table 1:** Metabolic function in macrophage subsets.

**Pathway**	**“Classically” activated macrophage**	**“Alternatively” activated macrophage**
Reactive oxygen species	Increased ROS production through mitochondrial ROS and NADPH oxidase	Mitochondrial ROS and NADPH oxidase activity minimal
Glycolysis	High aerobic glycolysis resulting in lactate production	Low glycolytic rate resulting in acetyl-coA production
Pentose phosphate pathway	Increased pentose phosphate pathway	Decreased pentose phosphate pathway
Tricarboxylic Acid Cycle	Fractured TCA cycle, broken at Idh and Sdh	Functional TCA cycle fed by acetyl-coA from beta-oxidation and glycolysis
Electron transport chain	Dysfunctional electron transport chain, resulting in mitochondrial ROS production	Functional electron transport chain resulting in ATP production
Fatty acid	Fatty acid synthesis from fractured TCA	Fatty acid beta-oxidation from lipoproteins
L-Tryptophan catabolism	L-Tryptophan catabolism by IDO results in suppression of aberrant inflammation	L-Tryptophan catabolism not induced
L-Arginine metabolism^*^	L-Arginine metabolism by iNOS resulting in nitric oxide production	L-Arginine metabolism by arginase resulting in L-ornithine and downstream metabolites

*ATP, adenosine triphosphate; Idh, isocitrate dehydrogenase; IDO, indoleamine-2,3-dioxygenase; iNOS, inducible nitric oxide synthase; NADPH, nicotinamide adenine dinucleotide phosphate; ROS, reactive oxygen species; Sdh, succinate dehydrogenase; TCA, tricarboxylic acid. Unless noted, references are in main text*.

* *Arginase 1 (Arg1) expression occurs along with iNOS in macrophages during intracellular infection, and is not sufficient on its own to define alternative activation*.

#### Glycolysis

Macrophage reliance on glycolysis to meet energetic demands has been demonstrated in several murine models of “classical” macrophage activation (88). Upon stimulation with cytokines or activation of PRR signaling, macrophages acquire a proinflammatory phenotype that correlates with robust upregulation of glycolytic pathways including hypoxia inducing factor alpha (HIF1α) (88). Activation of HIF1α induces transcription of hypoxic genes (e.g., glucose transporters, glycolytic genes) and IL-1β production by macrophages. Preferential skewing toward glycolysis favors proinflammatory macrophage effector functions as administration of the glucose analog, 2-deoxyglucose (2-DG), decreases macrophage inflammatory polarization, cytokine production and phagocytosis (88). Mice fed MCD diet and patients with NASH display increased hepatic macrophage HIF1α expression and exacerbated hepatic steatosis and inflammation (89). However, given the functional relevance of HIF1α to macrophage-mediated inflammation, additional studies are warranted to determine the impact of macrophage-intrinsic HIF1α in inflammation and NAFLD progression.

#### Pentose Phosphate Pathway

Pentose phosphate pathway (PPP) branches off glycolysis at glucose-6-phosphate, the second step in glycolysis. Through a series of dehydration and decarboxylation reactions, glucose-6-phosphate is converted to ribulose-5-phosphate. Macrophages upregulate the PPP in response to LPS, which yields two NADPH (used as cofactors for NOXs) and in turn promote inflammation and cellular damage (90–92). Additionally, the PPP is necessary for limiting the dissemination of various pathogens (93). In contrast, macrophages also use the PPP to resolve inflammation and ROS, as PPP results in glutathione reduction and subsequently maintains proper redox balance, limiting the consequences of extraneous ROS activity (94, 95). During hypercholesterolemia, cholesterol inhibits LPS-mediated PPP activity in inflammatory macrophages, leading to a foamy macrophage phenotype (96). Further, adipocytes are known to use NADPH metabolism to regulate lipid metabolism, and dysregulation of enzymes involved in the production of NADPH contributes to obesity and obesity-related pathology (97, 98). Despite the role of PPP in modulating inflammation, the contribution of this metabolic pathway to macrophage-intrinsic inflammation in the context of NAFLD is underdefined and requires in-depth examination.

#### Tricarboxylic Acid Cycle

Acetyl CoA, generated following glycolysis or beta-oxidation, enters the tricarboxylic acid (TCA) cycle, resulting in H_2_O, CO_2_, NADH and FADH_2_ generation. The latter two are utilized in the electron transport chain (ETC) to produce ATP. Despite the high energy yield of the TCA cycle, the process is time consuming and requires oxygen. In the context of a rapid inflammatory response, the TCA is downregulated and fractured in macrophages. Specifically, inflammatory macrophages reduce isocitrate dehydrogenase (Idh) and succinate dehydrogenase (Sdh) activity (90, 91). This results in decreased α-ketoglutarate formation but increased production of itaconate – a key anti-microbial metabolite. Accumulation of succinate in macrophages can inhibit the HIF1α-suppressing molecules prolyl hydroxylases (PHDs), allowing HIF1α to drive IL-1β and other inflammatory processes (90). Sdh is complex II of the ETC, which donates two electrons from FADH_2_ to produce the electron gradient that drives ATP synthesis. During LPS stimulation, the breakdown of Sdh feeds electrons through complex I, known as reverse electron transport, resulting in mitochondrial ROS production (99). The contribution of Idh and Sdh to NAFLD has not been investigated. However, it can be hypothesized that the hepatic inflammatory environment in NAFLD would drive the breakdown of the TCA cycle, as seen in LPS stimulated macrophages. Hence, additional studies are needed to formally address these postulates.

#### Fatty Acid Synthesis (FAS)

Excessive hepatocellular uptake of glucose is diverted to FAS pathways where glucose is converted into TGs and secreted to adipose tissue (AT) as VLDL (100). Under pathological conditions, *de novo* FAS by the liver is a primary cause of excessive hepatic steatosis (100). In contrast to glucose, insulin reduces AT lipolysis via suppression of hormone-sensitive lipase (HSL), thus regulating the circulation of FFAs in the periphery (100, 101). However, obesity-and NAFLD-associated insulin resistance limits HSL suppression, leading to increased AT lipolysis and FFA deposition in the liver (102). FAS is essential for immune cell proliferation in response to inflammatory insult. Macrophages upregulate FAS when undergoing “classical” activation. Monocyte treatment with macrophage colony stimulating factor promotes “classical” activation and expression of sterol regulatory element binding transcription protein 1c (SREBP1c), FAS target genes and increases lipid synthesis (103). Inhibition of SREBP1c reduces macrophage inflammatory capacity (104). Increased FAS drives KC inflammasome activation via nucleotide-binding oligomerization (NOD)-like receptor 3 (NLR3) signaling (103, 105). In fact, increased inflammasome activation has been observed in both murine experimental models and human NAFLD (105–107). Inflammasomes, reviewed elsewhere (105), are multiprotein complexes containing nucleotide-binding oligomerization domains NLRs. KCs are a key source of IL-1β and caspase 1, a critical NLR3 component that regulates downstream proinflammatory signaling (e.g., pro-IL-1β, pro-IL-18, ASC), and are elevated in livers from NASH patients (105, 108, 109). However, given the complexity of inflammasome signaling the underlying mechanism unique to macrophage inflammasome-driven inflammation in NAFLD, these processes warrant further examination. Additional studies are also needed to determine if targeted inhibition of FAS pathways in macrophages is sufficient to reverse inflammasome activation and subsequently improve NAFLD pathology.

#### Beta-Oxidation

Beta-oxidation of fatty acids (FA) is central for ensuring cellular and tissue energetic demands by breakdown and conversion of lipids into ATP. Under homeostatic conditions, fat storage and lipolysis are regulated in part by beta-oxidation. Members of the nuclear hormone receptor superfamily known as peroxisome proliferator activated receptors (PPARα, PPARβ, and PPARγ) are transcriptional modulators of beta-oxidation. PPARα, which is primarily expressed in the liver, has several endogenous ligands (e.g., FA, eicosanoids and other complex lipids) and acts as a master regulator for FA beta-oxidation (110, 111). Given the importance of hepatic steatosis in NAFLD and the relevance of beta-oxidation in immune responses, below we discuss the contribution of both mitochondrial and peroxisomal beta-oxidation to macrophage inflammation.

##### Mitochondrial beta-oxidation

Mitochondrial-beta oxidation breaks down short (< C_8_), medium (C_8_-C_14_) and long chain FA (C_14_-C_20_) (85). Long chain FA, which are a major component of the standard diet, are shuttled into the mitochondria via carnitine shuttles (carnitine palmitoyltransferase I) by linking with coenzyme A (acyl-CoA) (85, 112). Once acylcarnitine exchanges the carnitine molecule with CoA via exchange with carnitine palmitoyltransferase II the acyl-CoA proceeds into the beta-oxidation cycle. At the inner membrane the enzymatic activity of very long chain acyl-CoA dehydrogenase (VCLAD), shortens long chain acyl-CoAs. Shortened fatty acyl-CoAs are further oxidized by a trifunctional protein complex consisting of: Enoyl-CoA hydratase, 3-hydroxyacyl CoA dehydrogenase, and 3 ketoacyl CoA thiolase. Impaired beta-oxidation in macrophages prevents the degradation of lipids leading to the FA overload and rupture and release of toxic lipid species (85). Although, the traditional view is that FA beta-oxidation is essential for polarization of “alternatively” activated macrophages, recent evidence suggest that inhibition of this pathway may in fact promote a “classical” macrophage phenotype. Etomoxir driven inhibition of beta-oxidation or knockdown of CPT-1 results in reduced fatty acid oxidation (FAO) but increased proinflammatory signaling, cytokine production, ER stress and ROS levels (113–115). Thus, additional studies are needed to formally determine how mitochondrial FAO impacts macrophage inflammation and NAFLD progression.

##### Peroxisomal beta oxidation

Oxidation of very long chain fatty acids (VLCFAs) (C>_21_) is exclusive to the peroxisome due to the selective presence of very long chain acyl-CoA synthetase (85). Aside from VLCFAs, long chain dicarboxylic acid, eicosanoids, and bile acid precursors are also oxidized within the peroxisomes. Compared to mitochondria, 3 enzymes Acyl-CoA oxidase 1 (Acox1), enoyl CoA hydratase/L-3-hydroxyacyl CoA dehydrogenase bifunctional protein and 3-ketoacyl CoA thiolase regulate peroxisomal beta-oxidation (85). Whole body *Acox1* null mice and *Acox1*^*Lampe*1^ mice, which features a point mutation rendering the *Acox1* gene inactive, spontaneously develop steatosis and steatohepatitis (85, 116). *Acox1*^*Lampe*1^ mice also exhibit increased systemic inflammation both at baseline and after LPS challenge *in vivo*, increased hepatic expression of macrophage recruiting chemokines and macrophage infiltration into the liver following short term high fat diet (HFD) feeding. HFD feeding combined with secondary LPS insult *in vivo* further exacerbates liver pathologies in *Acox1*^*Lampe*1^ mice (86). Together, these data suggest that peroxisomal beta-oxidation regulates macrophage-intrinsic inflammation and NAFLD pathogenesis. However, the underlying processes by which peroxisomal beta-oxidation regulates macrophage function and inflammation remain understudied. Similarly, the contribution of peroxisomal beta-oxidation to inflammatory potential of other immune cells and their contribution to NAFLD progression remains poorly understood.

#### Amino Acid Metabolism

Amino acids (AA) are critical precursors for several metabolic pathways. For example, glutamine and aspartate are necessary for purine and pyrimidine synthesis as well as feeding the TCA cycle via α-ketoglutarate production. Valine and leucine fuel the synthesis of branched chain FA. Direct metabolism of tryptophan (L-TRP) and arginine (L-ARG) by macrophages and other myeloid cells regulate key immunologic processes. For this reason, below we focus on how metabolism of tryptophan and arginine within macrophages modulates their inflammatory potential.

##### Tryptophan

In macrophages, L-TRP metabolism is tightly regulated by two isoforms of the enzyme indoleamine 2,3-dioxygenase (IDO1 and IDO2). IDO is the rate limiting enzyme that converts L-TRP into N-formylkynurenine (117). Early reports focused on L-TRP depletion as the central mechanism of immune modulation, yet more recent literature deemphasizes L-TRP depletion and reports kynurenine production as the key regulator of immune responses (117, 118). The downstream products of kynurenine modulate immune responses to infection, cancer, and autoimmune diseases (117, 119, 120). IDO activity is induced in macrophages following IFNγ, LPS, or TNF stimulation and can be further enhanced with IL-1β co-stimulation (121–125). Several studies have begun to address the contribution of IDO-mediated L-TRP metabolism during NAFLD. Kynurenine is increased in the serum of obese subjects, and IDO1 is upregulated in the liver and WAT in obesity (126). IDO-deficient mice displayed elevated liver fibrosis, increased hepatic macrophage infiltration, and higher concentrations of IL-1β, IL-6, and IFNγ in obesity. However, these mice are protected from HFD-driven weight gain, hepatic steatosis, and oxidative stress (127). Interestingly, mice lacking IDO1 expression in macrophages and neutrophils exhibited normal weight gain and insulin sensitivity in obesity (128). Mice with an intact bone-marrow derived immune system, but lacking IDO1 in all other tissues, displayed a similar protection from HFD-induced metabolic disease as the mice with global IDO1 deletion. In sum, these data suggest that IDO contributes to multiple aspects of NAFLD progression and that non-hematopoietic IDO activity may play a key role in regulating NAFLD pathogenesis. Follow-up studies are needed however to dissect the contribution of IDO activity during the full spectrum of NAFLD pathogenesis and to evaluate IDO activity within radiation-resistant tissue macrophages.

L-TRP supplementation has been explored in multiple studies. Mice fed a high fructose diet exhibit reduced liver weight and hepatic lipid accumulation when supplemented with L-TRP (129). Clinical studies examining patients with hepatic steatosis or NASH found supplementation, twice daily, with L-TRP for 14 months result in decreased plasma LDL, TG, and gamma gluthamylo transpeptidase levels with a correlative decrease in plasma IL-1β, IL-6, and TNF (130, 131). Key studies, however, are needed to identify where supplemental L-TRP and its downstream metabolites accumulate, and whether L-TRP is available for IDO activity within the diseased liver. Considering the availability of conditional IDO1 knockout mice (128), it is now feasible to separate the contribution of IDO activity by macrophages as well as other immune and non-immune cell types during the initiation and progression of NAFLD. These, studies would help determine how IDO modulates immune responses and NAFLD pathogenesis.

##### Arginine

Historically, macrophage polarization was characterized in part by the ability to metabolize L-ARG. “Classically” activated macrophages upregulate inducible nitric oxide synthase (iNOS) which converts L-ARG to L-citrulline and anti-microbial nitric oxide (NO). In contrast, “alternatively” activated macrophages upregulate arginase 1 (Arg1) to metabolize L-ARG into ornithine and urea (132, 133). It is now appreciated that Arg1 and iNOS expression can occur within similarly stimulated macrophages, adding to the complexity of defining “classical” and “alternative” macrophage activation profiles (134–136). Regardless, macrophage L-ARG metabolism has been documented to restrict intrinsic and extrinsic immune cell function. Blocking arginase activity or eliminating Arg1 within macrophages allows for increased L-arginine availability for NO production and anti-microbial activity but can also be associated with unrestricted lymphocyte activity and increased tissue pathology (132, 136–141). Thus, understanding how L-ARG metabolism is regulated during altered inflammatory and metabolic states, including NAFLD, is of considerable interest.

Limited studies have focused on the contribution of enzymes involved in the breakdown of L-ARG in NAFLD. Mammals possess two arginase isoforms (Arg1, Arg2) that are differentially expressed within tissues. The importance of macrophage Arg1 in NAFLD has not been addressed. The role of Arg2 in NAFLD, despite published studies, remains undefined. Opposing findings employing Arg2-deficient mice have shown that Arg2-deficiency results in development of spontaneous hepatic steatosis and increased liver injury (142) or promotes decreased NAFLD severity in obesity (143). Although NO contributes to NAFLD, additional studies are needed to determine the critical source of NO as NO inhibitors have various specificities and differ in their ability to regulate disease severity (144). L-ARG supplementation has also been shown to reduce NAFLD severity (145). As such, studies determining the contribution not only of L-ARG utilization, but also of L-ARG synthesis during NAFLD are warranted. The necessity of L-ARG synthesis within macrophages has recently been described during infection, suggesting extracellular L-ARG is not available in sufficient concentrations to drive effective macrophage function (135, 146, 147). Accounting for the considerable influx of inflammatory macrophages in NAFLD, future studies aimed at addressing macrophage-specific modulation of L-ARG metabolism with existing molecular tools (e.g., *Arg1*^flox^, *Asl*^flox^, Nos2-deficient) (148–150) will be necessary to dissect how various macrophage populations manipulate the liver microenvironment and NAFLD progression.

#### Macrophages and Trace Metals

Trace metals including iron, zinc and copper are essential for many cellular functions and for optimal adaptive and innate immune responses (151). Among these three metals, iron and copper exert an important influence on the genesis of NAFLD (152–155). Adults, but not children, with NAFLD manifest increased circulating concentrations of ferritin; however, both age groups exhibit increased transferrin saturation (153, 154). Excess iron accrual in the liver, specifically KCs (156) is associated with elevated amounts of hepcidin, which blocks iron egress mediated by ferroportin. Hepcidin binds to ferroportin and enhances its degradation. The accumulation of iron promotes “classically” activated macrophage polarization and production of proinflammatory cytokines that enhance the inflammatory response (157, 158). Excessive iron accumulation in both KCs and hepatocytes is associated with NAFLD (152). Inflammatory mediators, induced by lipid overload, drive increased hepcidin and decreased iron export from KCs and hepatocytes, in turn exacerbating NAFLD severity. Although copper is connected to iron homeostasis, the former metal is diminished in patients with NAFLD. Copper deficiency is associated with a decrease in ceruloplasmin ferroxidase which promotes iron release (152, 159). Aside from the effects of copper on iron regulation, the paucity of copper would reduce generation of copper/zinc superoxide dismutases that scavenge ROS and subsequently impairing the cellular defenses to ROS-mediated damage. Despite the knowledge of low zinc concentrations in chronic liver disease and damage (160) the role of zinc in NAFLD has not been investigated. How zinc deficiency augments liver damage is not well defined. Experiments determining the necessity of superoxide dismutases to combat excess ROS would provide valuable insight but have yet to be performed.

### Adipose Tissue/Adipocytes and NAFLD

The traditional perspective of AT was that of a simplistic fat storing/releasing organ playing a role in energy homeostasis (161). It is now well-appreciated that AT is a highly metabolic, active and plastic organ comprised of various types of cells (e.g., adipocytes, progenitor stem cells within the stromal vascular fraction, endothelial cells, fibroblasts, and immune cells) (162, 163). The AT regulates the accommodation of excess energy through storage of circulating dietary lipids and *de novo* lipogenesis. In the case of nutrient shortage, lipolysis controls/regulates the release of hydrolyzed TGs as glycerol and FFAs to provide energy to surrounding tissues (164–166). Obesity-associated changes in AT robustly modify AT function including production of hormones, cytokines and adipokines. Which cells within AT tissue contribute to such changes and how the low-grade systemic and AT inflammatory state in obesity/metabolic disease impacts AT function is poorly understood.

Adipose tissue macrophages (ATMs) are believed to play a major role in regulating AT inflammation. In general, healthy AT, is believed to contain a balance between “alternatively” and “classically” activated ATMs. In contrast, the unhealthy/inflammatory AT, houses an increased number of “classically” activated ATMs. “Classically” activated ATMs produce an array of proinflammatory mediators (e.g., IL-6, TNF, IL-1β, IFNγ) that further amplify AT inflammation and promote additional macrophage and other immune cell recruitment and activation. Cumulatively, such events, in obesity, fuel a chronic low-grade inflammation within the AT (167, 168). In addition to the enhanced release of soluble mediators, AT inflammation drives expression of integrin α4 on macrophages and vascular cell adhesion molecule 1 (VCAM-1) on adipocytes allowing for AT macrophage accrual (43). The inhibition of integrin α4 reduces ATM retention and AT inflammation. Notably, individuals with NAFLD exhibit high expression of VCAM-1 in AT underlining the importance of this cell-cell interaction pathways (169).

**Figure 3:**
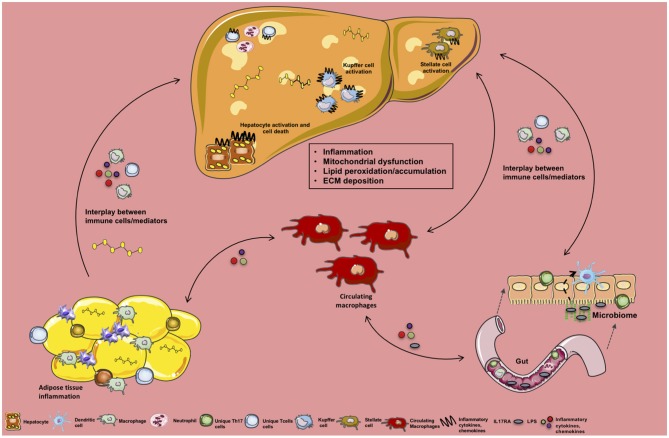
Crosstalk between tissue-specific inflammation and macrophage function in NAFLD. Schematic overview of the crosstalk between various organs, their specific immune cells and inflammatory mediators during NAFLD. Obesity-associated low-grade, chronic inflammation and altered gut microbiome impacts immune cell crosstalk between the gut, circulating monocytes/macrophages, and the liver. In addition, obesity-associated adipocyte expansion promotes hypoxia leading to adipose tissue (AT) inflammation, activation of adipose tissue macropahes (ATMs) and fuels infiltration of various immune cells and inflammatory mediator production (e.g., FFAs, ROS, cytokines, chemokines) to be sensed by circulating macrophages and hepatocytes. Collectively these processes alter hepatocellular lipid metabolism, contributing to steatosis and proinflammatory cytokine (IL-17, TNF, IFNγ, IL-6) and chemokine production (CCL2, CXCL9, CXCL10). Moreover, this inflammatory state activates hepatic stellate cells (HSCs) and Kupffer cells (KCs) in turn contributing to extracellular matrix deposition (collagen fibers) and progression to fibrosis.

Adipocytes play a pivotal role in metabolic disease by promoting chronic inflammation via release of FFAs in response to increased circulating levels of TNF. These FFAs translocate to the liver and skeletal muscle propagating inflammation and insulin resistance (170, 171). Mechanistically, TNF inhibits PPARγ (172) and CCAAT/enhancer binding protein (C/EBPα) in adipocytes that is needed for the expression of adipocyte-specific GLUT4 and insulin receptor (IR) to maintain insulin sensitivity (173, 174). This suggests that like ATMs, adipocytes themselves play an important role in maintaining AT metabolic processes. Recent studies demonstrate that adipocytes, like immune cells, exhibit immune-like potential (175, 176). Specifically, adipocytes express various innate immune receptors including RIG-I-like receptors (RLR), NLRs, and nucleotide oligomerization domains (NODs) (177, 178). NOD-1 signaling suppresses adipocyte differentiation and contributes to induction of the NF-kB (177, 179). Adipocyte sensing of various PAMPs leads to production of multiple inflammatory mediators (e.g., cytokines, chemokines, adipokines) (180). In obesity, the main mechanisms associated with unlocking adipocyte-intrinsic inflammation are: (a) obesity-associated endotoxemia and (b) AT hypoxic micro-environment which leads to ER stress, inflammatory cytokine production, cell death, release of lipid content and debris and induction of the inflammatory mediators (181). Adipocyte production of inflammatory mediators is potentially sensed by ATMs and leads to their activation (161, 168, 180, 182). Adiponectin, an adipokine, exerts either anti- or pro-inflammatory effects on macrophages. It inhibits macrophage functions (e.g., phagocytosis, cytokine production) and induces proliferation of “alternatively” activated macrophage in AT (183–185). Conversely, adiponectin also induces pro-inflammatory signaling cascades through NF-kB activation and upregulation of pro-inflammatory cytokines (e.g., TNF-α, IL-6, and IL-8) (186, 187). However, detailed analysis of specific adipocyte mediators and adipokines relevant to altered ATMs polarization and activation is needed. Similarly, whether obesity-activated adipocytes or ATMs directly play a role in NAFLD pathogenesis is not fully understood and should be further investigated. A brief summary of the above discussed processes is depicted in Figure 3.

### Therapies

Ultimately, the improvement of experimental models to more closely recapitulate human NAFLD would be ideal for the discovery and development of therapies targeting various metabolic pathways in macrophages. Diet and lifestyle changes help in reversing NAFLD progression. Thus, it is not surprising that several pharmacological drugs that target metabolic and inflammatory and molecular mechanisms important in NAFLD progression are currently being examined (188). Hepatic lipid accumulation is an initial driver of NAFLD pathogenesis (9, 84). Intuitively, use of therapeutic drugs that target lipid metabolism is actively pursued. Glitazones, which are a class of insulin-sensitizing drugs, are effective in regulating lipid metabolism. They increase FAS and FA uptake by adipocytes, thus increasing lipid loading in AT instead of ectopic organs (e.g., liver and muscle) (189, 190). However, due to their association with increased risks of heart failure the use of glitazones as a treatment option has not been pursued in the clinic (191, 192). Sodium glucose co-transporter 2 (SGLT2) inhibitors have also proven efficacious in regulating NAFLD-associated dyslipidemia by inhibiting hepatic expression of lipogenic genes (e.g., sterol regulatory-element binding protein 1-c, fatty acid synthase, acetyl-CoA carboxylase 1, and sterol CoA desaturase), hepatic macrophage infiltration and expression of inflammatory cytokine production (190, 193, 194). However, underlying mechanisms that govern this process remains an area of investigation. Upregulation of oxidative stress, inflammation and apoptosis pathways are associated with NAFLD pathogenesis. NASH patients display increased hepatic activation of apoptosis signal-regulating kinase 1 (Ask1). Activation of Ask1 by TNF causes oxidative and ER stress, and induction of p38 and JNK signaling (188, 190, 195). Ask1 inhibition reduced hepatic steatosis, inflammation and fibrosis (196, 197). However, given that phenotypical outcomes of Ask1 inhibition in mice are not often recapitulated in humans, more effective “humanized” mouse models are needed (198, 199). Further, the effects of Ask1 inhibition on macrophage inflammation in NAFLD pathogenesis remains underdefined. Limiting the detrimental effects of obesity-associated microbiome alteration and subsequent systemic endotoxemia which contribute to NAFLD pathogenesis is another active area of investigation for drug development. Excessive PRR activation and inflammation resulting in liver injury is characteristic of NAFLD. JKB-121, a TLR4 antagonist, prevents LPS induced inflammatory liver injury in MCD diet models of NAFLD. However, given that obesity modulates the expression of multiple TLRs, more studies are needed to determine the impact of ablation of other TLRs in NAFLD pathogenesis. In addition, there are several other therapeutic approaches regarding use of ACC inhibitors, fructose inhibitors and obetocholic acid inhibitors (188, 190) for the treatment of NAFLD. In sum, several potential avenues for NAFLD therapies are being pursued. Specifically, there is a need for studies to allow for HFD-driven induction of hepatic fibrosis (41), eliminate gender bias by employing a more “human”-like disease state (e.g., thermoneutrality) (200), CCL4 experimental models of fibrosis (201) use of various murine strains/genotypes to mimic genetic diversity as well as expansion of such findings into non-human primate models of NAFLD (198). Use of such wide ranging experimental models would be beneficial in the development of therapeutic targets that may prove more effective in the clinic. Thus, in sum, given the interplay between metabolism and inflammation, additional therapies targeting macrophage polarization, chemo-attracting, inflammatory and metabolic pathways are needed—something that may be achieved by improving experimental modeling of disease.

## Conclusion

Overall, this review highlights the inflammatory processes associated with macrophage polarization, tissue recruitment and inflammation and the role of such processes in NAFLD. We also extensively discuss how cellular metabolic pathways may contribute to macrophage-driven inflammation. Given the metabolic changes in obesity and inflammation the potential benefits to be gained from understanding the interplay between various metabolic and inflammatory pathways in macrophages are immense. Further elucidation of the crosstalk between macrophages and other tissues/immune cells similarly remains an exciting area of exploration. However, subsequent to the detailed interrogation of the afore discussed cellular and molecular processes in NAFLD, validation of such processes in multiple experimental models of NAFLD will be required.

## Author Contributions

JO, MM, MM-F, MD, GD, JQ, and SD wrote the manuscript. All authors have reviewed the manuscript and approve the final version.

### Conflict of Interest

The authors declare that the research was conducted in the absence of any commercial or financial relationships that could be construed as a potential conflict of interest.
